# The Impact of the COVID-19 Pandemic on the Prognosis of Laryngeal Adenoid Cystic Carcinoma: A Case Report and a Literature Review

**DOI:** 10.3390/diagnostics13050905

**Published:** 2023-02-27

**Authors:** Irene Fatuzzo, Andrea Colizza, Piero Giuseppe Meliante, Haitham Elfarargy, Roger Altomari, Marco Fiore, Massimo Ralli, Daniela Messineo, Antonio Greco, Marco de Vincentiis, Christian Barbato, Antonio Minni

**Affiliations:** 1 Department of Sense Organs DOS, Sapienza University of Rome, Viale del Policlinico 155, 00161 Roma, Italy; 2 Department of Otorhinolaryngology, Faculty of Medicine, Kafrelsheikh University, El-Geish Street, Kafrelsheikh 33155, Egypt; 3 Institute of Biochemistry and Cell Biology (IBBC), National Research Council (CNR), Department of Sense Organs DOS, Sapienza University of Rome, Viale del Policlinico 155, 00161 Roma, Italy; 4 Department of Radiology, Oncology, and Anatomopathological Science, Sapienza University of Rome, 00161 Rome, Italy; 5 Division of Otolaryngology-Head and Neck Surgery, Ospedale San Camillo de Lellis, ASL Rieti-Sapienza University, Viale Kennedy, 02100 Rieti, Italy

**Keywords:** laryngeal adenoid cystic carcinoma (LACC), COVID-19, laryngectomy, adenoid cystic carcinoma (ACC), head and neck cancer

## Abstract

Laryngeal adenoid cystic carcinoma (LACC) is a sporadic neoplasm, especially if supraglottic. The COVID-19 pandemic worsened the presenting stage of many cancers and impacted their prognosis negatively. Here, a case of a patient with adenoid cystic carcinoma (ACC) with delayed diagnosis and a rapid deterioration with distant metastasis due to the COVID-19 pandemic is illustrated. Next, we present a literature review of this rare glottic ACC. The COVID-19 pandemic worsened the stage of presentation of many cancers and adversely affected their prognosis. The present case had a rapidly lethal course, undoubtedly due to the diagnosis delay caused by the COVID-19 pandemic, which impacted the prognosis of this rare glottic ACC. Strict follow-up is recommended for any suspicious clinical findings, as an early diagnosis will improve the disease prognosis, and to consider the influence of the COVID-19 pandemic, especially on the timing of common diagnostic and therapeutic procedures for oncological diseases. In the post-COVID-19 era, it is important to generate new diagnostic scenarios to achieve an increasingly rapid diagnosis of oncological diseases, especially the rare ones, through screening or similar procedures.

**Figure 1:**
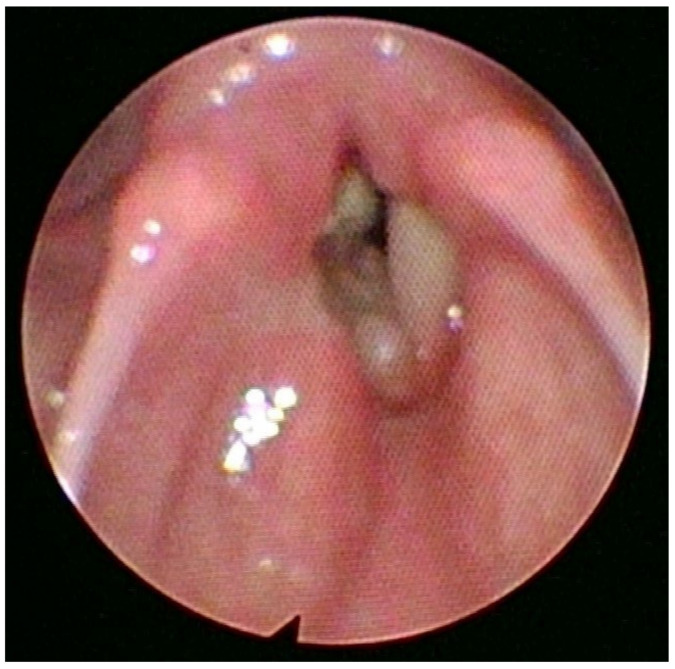
Endoscopic view of the larynx showing the lesion. The adenoid cystic carcinoma (ACC) arises from the minor salivary glands. It accounts for 1–5% of all head and neck malignancies. Since the minor salivary glands are present in small amounts throughout the larynx, the laryngeal adenoid cystic carcinoma is sporadic, representing less than 1% of all laryngeal malignancies [1]. Regarding the onset of laryngeal ACC (LACC), the prevalent age ranges from 50 to 60 years. However, younger generations can be affected, and both sexes are equally affected, with a slight male predominance and a male-to-female ratio of 1,5:1. There is no evidence connecting LACC etiology with smoking. An early perineural and hematological spread make this kind of carcinoma liable for local recurrence and distant metastasis, especially to the lung. Therefore, is important to increase the frequency of controls during follow-up [2]. Laryngeal ACC can originate from any part of the larynx. The most common origin is the subglottic area (64%), followed by the supraglottic area (25%), the glottic area (5%), and the trans-glottic area (6%) [3,4]. The clinical presentation is usually variable and related to the lesion location [5,6,7]. In November 2021, a 70-year-old no-smoker female patient presented to our hospital’s emergency department with stridor, severe dyspnea at rest, and hoarseness of voice. The O2 saturation level was 87% on air without cyanosis. An endoscopic laryngeal examination revealed bilateral vocal cord paralysis in adduction. Firstly, the patient underwent an urgent tracheostomy under general anesthesia. The procedure also included a laryngeal examination (micro-laryngeal surgery) under general anesthesia with tumor mapping, which revealed a bilateral mucosal thickening of the anterior thirds and anterior commissures of both vocal folds and a right vocal fold submucosal thickening (Figure 1). Multiple biopsies from different laryngeal areas were taken for histopathological examination. The pathological tissue revealed the presence of an adenoid cystic carcinoma of the solid type associated with the immunophenotype CK AE1 AE3 +, CD117+, CK7+/−, p63 +/−, p40+/−, Vimentin +/−, SMA+/−, S100+/−. Then, the patient underwent a CT scan of the neck chest and brain with a contrast medium and an abdominal ultrasound examination. The first chest, brain, and abdominal radiological evaluations did not show metastatic lesions. The neck CT scan revealed the presence of small submucosal bilateral glottic masses, associated with increased cervical lymph nodes volume, without subglottic and extra-laryngeal extensions (Figure 2).

**Figure 2:**
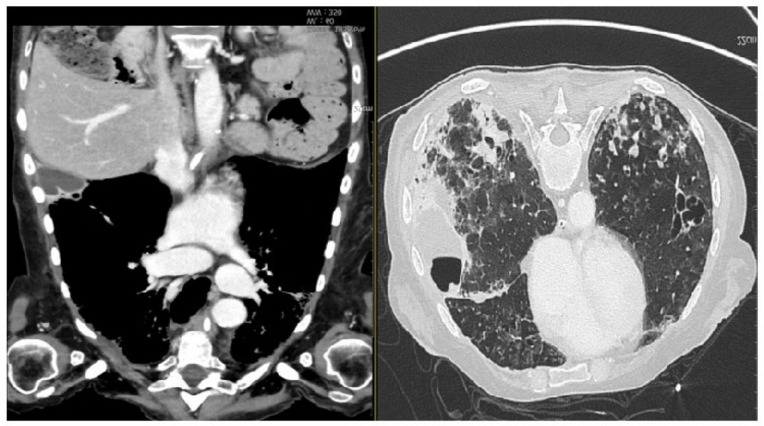
CT scan showing (during hospitalization) pneumonia by Cytomegalovirus. At the lateral-basal segment of the right inferior lobe, in the subpleural level, the gross intraparenchymal collection with hydro-aerial content is compatible with the pneumatocele. In the lung window, multiple areas of parenchymal thickening are visible in the right basal location. (On the left, coronal section parenchymal window, on the right, axial section lung window). Partial laryngectomy (OPHL II B) with bilateral selective neck dissection (cervical nodal Robbins levels II–IV) and postoperative radiotherapy were planned to manage this case. Unfortunately, this planned surgical intervention was impossible because of the patient’s poor general conditions. Moreover, one of the follow-up CT scans of the chest (three months after the initial one) revealed the presence of bilateral diffuse multiple micronodules, which were considered early distant metastasis from the laryngeal adenoid cystic cancer (Figure 3).

**Figure 3:**
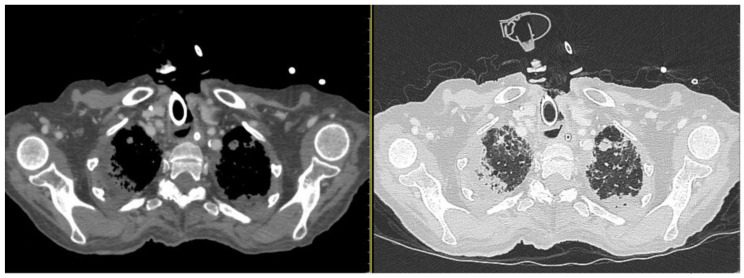
CT scan windows showing metastasis and inflammatory phenomena on both sides of the lung. (On the left, axial parenchymal lung section. On the right, axial lung window). During hospitalization, bacterial pneumonia began, worsened by secondary viral (Cytomegalovirus) and fungal pneumonia. The patient received a triple antibiotics course with an antiviral, an antifungal, and systemic and local inhalational corticosteroids. However, despite the medical therapy, the chest condition deteriorated progressively (Figure 4).

**Figure 4:**
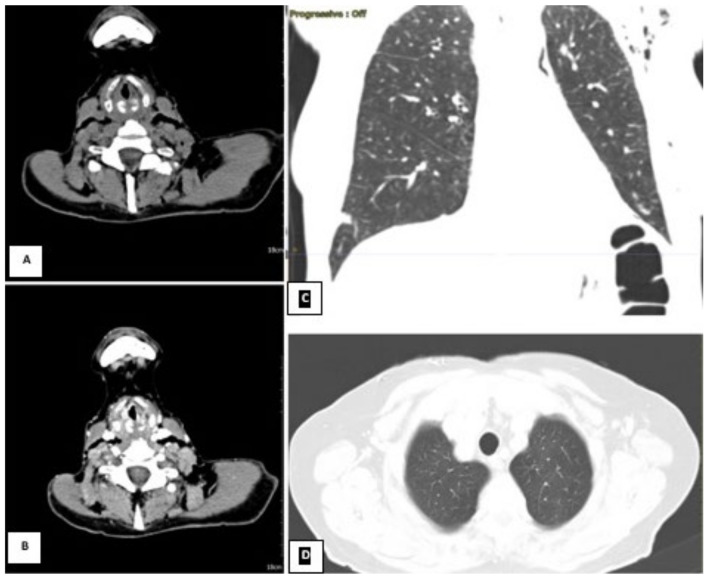
CT scan on the left. The neck scan revealed the presence of small submucosal bilateral glottic masses, associated with increased cervical lymph node volume, without subglottic and extra laryngeal extensions. (**A**) Axial section basal CT scan on the left. (**B**) Axial contrast-enhanced CT on the right. Lung window showing a small scar at the level of the right lower lobe from previous pneumonia reported by the patient; (**C**) coronal and (**D**) axial lung window). Due to the worsening conditions, the patient was mechanically ventilated because of acute respiratory failure. Despite the therapy and mechanical ventilation, the pulmonary functions deteriorated progressively, resulting in the patient’s death. The patient was not diabetic, hypertensive, or cardiopathic and was not a smoker. By anamnestic clinical history, we discovered that two years before this event (October 2019), the subject presented mild hoarseness of voice. At the time, an endoscopic laryngeal examination revealed bilateral mobile vocal folds without apparent abnormalities. For further confirmation, the subject underwent a laryngeal exam under general anesthesia, which showed the absence of any macroscopic lesion, and the histopathological results of the biopsies were negative. The physicians scheduled a follow-up after three months, but unfortunately, the lockdown caused by the COVID-19 pandemic and the fear of viral infection prevented her to attend the recommended follow-up visits. The patient was COVID-19-negative throughout the whole illness (Table 1). We performed a literature analysis by searching the PubMed database for ‘laryngeal adenoid cystic carcinoma’. We did not limit the search to article types because of the rarity of the disease and the little number of papers about it. We choose only papers published in English within the past five years. The articles in the database whose full text could not be found were also excluded. The title and abstracts of the identified manuscripts were initially screened and selected by all authors independently (IF, AC, PGM, HE, RA, MF, MR, DM, AG, MdV, CB, and AM) based on their relevance to the review topic. The following set of shared chosen inclusion criteria was applied individually to the selected articles in their full-text version: primary laryngeal affection of adenoid cystic carcinoma and therapy consensus of LACC. The literature search yielded 48 papers. Subsequently, 28 studies were excluded because they did not meet the objective of our review, and 20 studies were included and discussed (Figure 5 and Table 2).

**Table 1:** Clinical synopsis of the laryngeal adenoid cystic carcinoma.

**Clinical timeline**
Hoarseness without laryngeal mucosal abnormalities in 2019. Admission to the emergency room in 2021. Tracheostomy.
**Clinical staging**
Laryngeal biopsies and diagnosis of ACC. CT scan with contrast. Partial laryngectomy with bilateral selective neck dissection planning. Clinical worsening. Death.

**Figure 5:**
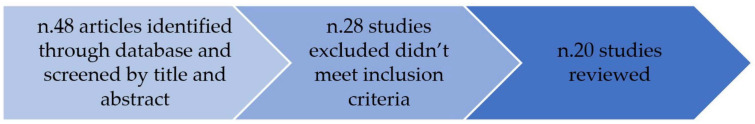
Articles selection on laryngeal adenoid cystic carcinoma. Primitive LACC is a rare head and neck carcinoma with slow growth but a high rate of malignancy due to its frequent perineural invasion. A high percentage of distant metastasis has been reported both at first diagnosis and during follow-up. The main distant metastasis site for LACC is the lung, but metastasis can develop in many sites. Iype et al. even described a case of an isolated scapular metastasis [8]. Although the first evaluation of neoplastic disease usually involves TNM staging according to AJCC, which is considered to have a significant prognostic value, Taha M. et al. observed that this is not true for LACC [9]. The histologic grade seems to be a more significant prognostic factor for survival in the presence of LACC, although this finding was established in a small sample of patients [10,11,12,13]. Radiation therapy is not considered a primary curative treatment for adenoid cystic carcinoma, whatever its location, but it has been widely used as an adjuvant treatment. Benefits in terms of local control and survival with adjuvant radiotherapy have been reported in many papers [13].

Tan et al. [14] tried to create an overview of rare tumors of the larynx and of therapeutic protocols approved for laryngeal adenoid cystic carcinoma. It seems that there are no international guidelines for their treatment, but only recommendations, as reported in Lionello’s work [15]. The recommended treatment for LACC is extensive surgical resection combined with postoperative radiotherapy. However, there is no agreement regarding the treatments for LACC. According to the AJCC criteria, surgery seems to be the best therapeutical choice, with a good disease-free survival rate in the following 5 years; however, the outcomes are not satisfactory [14,15,16]. Total laryngectomy was the most used surgical procedure [1,12,17,18]. However, nowadays the best surgical treatment is chosen based on the staging of the disease, thus considering surgical approaches aimed at preserving the morphology and function of the larynx [19]. The main goal is to free patients from cancer and at the same time guarantee them the best quality of life. Iandelli et al. reported only two cases of laryngeal non-SCC and argued that conservative surgery is possible without affecting the patients’ survival [20]. As the conservative approach is the best choice, Kozhanov et al. reported a case of endolaryngeal resection of ACC in a T1N0M0 R0 [21]. Partial laryngectomy could be a valid alternative to radical surgery according to disease staging [22]. For example, Wang et al. reported a partial laryngectomy for LACC, and the patient had no evidence of disease recurrence or metastasis during the follow-up period [23]. To improve the outcome, adjuvant therapy is to be considered in case of adverse features such as close or positive margins, T3-4, neural and perineural invasion, and lymph node metastases. Some authors proposed concurrent chemotherapy and radiotherapy, such as Vardaxi et al. [24]. No studies compared these different therapeutic protocols. Even though LACC is relatively radioresistant, Akbaba et al. stressed the role of RT as an alternative to total laryngectomy [25]. The same group reported eight cases of LACC who underwent radiotherapy with carbon ions (C12) at the Heidelberg Ion Beam Therapy Center (HIT). The small number of enrolled patients does not allow us to come to conclusions [26]. Adjuvant radiotherapy is the best choice after surgical treatment and should be used in each case of perineural invasion, as also Marchiano et al. observed in 2016 [27]. Cui et al. reported, in 2019, that radiant therapy after surgery is more recommended, but there are many cases of recurrent LACC with or without distant metastasis in the lung some years after treatment [1]. The adenoid cystic carcinoma represents a rare tumor originating from the salivary glands that can affect the upper aero-digestive tract, including the larynx. It manifests with a slow growth pattern, often with macroscopic submucosal characteristics, such that it does not manifest symptomatically until advanced stages. Based on the laryngeal subsite involved, there are different clinical manifestations. In each case, the severity of the pathology is assessed according to both clinical and radiological analyses, but a definitive histological staging is usually necessary. Therefore, to determine its local extension, the histological pattern should always be identified, with particular attention to perineural invasion that characterizes high-risk cancers. After a careful evaluation of the literature about LACC, we can state that the first choice of treatment should be the most radical surgery as possible, considering conservative procedures such as partial laryngectomies among the options. The recommendation for adjuvant treatment is required on a case-by-case basis depending on the histology, extent, and stage of the pathology. A considerable number of distant metastases was found during follow-up. It should be emphasized that there is no direct correlation between the degree of pathology severity and the incidence of distant metastasis. Considering our clinical case, when the patient came to our clinic for the first time two years earlier, she had a recent onset of mild voice hoarseness. Although the endoscopy examination revealed no lesions, with bilateral mobile vocal folds without apparent mucosal abnormalities or neck involvement, we tried to confirm the exclusion of any malignant lesion by a complete laryngeal examination under general anesthesia. The negative clinical, radiological, and histopathological results denied the proposal of a suspicious malignancy. This negativity during the pathology’s starting period was most likely due to the inert submucosal nature of LACC lesions, which may take years to be noticeable. However, for additional confirmation, we asked the patient to follow a restricted follow-up protocol and to undergo an endoscopic office laryngeal examination every three months for the early detection of any developing lesion. Unfortunately, the patient did not follow this protocol and two years later developed a sudden onset of stridor which necessitated tracheostomy due to a T3 glottic LACC lesion. This follow-up delay was mainly due to the lockdown during the COVID-19 pandemic. After diagnosing the glottic T3 N0 M0 ACC lesion, we decided on a therapeutic plan in the form of partial laryngectomy (OPHL II B) surgery with neck dissection, with adjuvant radiotherapy. We decided on a partial laryngectomy to preserve the voice function and reserved total laryngectomy for recurrence. ACC is conventionally thought to be radioresistant. However, some studies have shown prolonged patient survival and decreased recurrence following radiotherapy, suggesting that ACCs are radiosensitive [28]. Moreover, postoperative adjuvant radiotherapy may help confirm negative free margins after surgery, as this tumor has a high liability for local growth because of perineural and hematological spread. This therapeutic plan was canceled because of a pulmonary infection and the bad general conditions of the patient. In addition, the presence of a cancer lesion decreased her immune function, resulting in refractory pneumonia. Moreover, this effect was worsened by the following uprising of lung metastasis from the laryngeal ACC lesion [28]. During the COVID-19 pandemic, all medical services were shortened, and most outpatient clinics and services were canceled. The medical services were mainly directed to manage COVID-19 cases and control the pandemic. In addition, the main priorities were the emergency cases and the oncological surgeries. All these circumstances were associated with a public fear of seeking medical services to avoid COVID-19 infection, which was associated with high mortality rates [29,30,31]. This worsened the presenting stage of many cancers and impacted their prognosis badly. According to the research of Stevens et al., head and neck mucosal squamous cell carcinoma patients presented with more advanced clinical nodal disease during the early months of the COVID-19 pandemic, despite no change in the symptoms [32]. Moreover, Tevetoğlu et al. concluded that the COVID-19 pandemic caused delays in diagnosing and treating many diseases, such as head and neck cancers. Admissions with advanced-stage disease and the need for more complex reconstructive procedures increased [33]. The disease-specific survival rates of LACC are 69% and 49% at 5 and 10 years, respectively, after a primary treatment [15]. In contrast, the presenting case had a rapidly progressive disease course and died without receiving oncological management. Many factors were responsible for this bad prognosis, such as the delay in the diagnosis, the advanced stage at presentation, the bad general conditions, and the presence of distant metastasis to the lung. This case report shows that the COVID-19 pandemic worsened the stage of presentation of many cancers and adversely affected their prognosis [34]. The management of cancer patients was deeply modified, and oncology departments’ staff reorganized their protocols and priorities to counteract the COVID-19 impact. The presence of a COVID-19 infection in cancer patients increased the complexity of cancer treatment and the risk of complications [35]. In most patients, elective cancer treatments were postponed, with the aim of finding a balance between a potential COVID-19 infection and cancer treatment protocols in the cancer population [35]. The present case of LACC had a rapidly lethal course, undoubtedly due to the effects of the COVID-19 pandemic, which affected the prognosis of this rare glottic ACC. We recommend rigorous follow-up for any suspicious laryngeal clinical findings. However, it is not yet established that early intervention can improve the prognosis of laryngeal lesions. Overall, the purpose of our work is to recommend to strictly apply the already-known diagnostic–therapeutic algorithms and to alert patients about their clinical conditions. It would also be desirable to try to think of screening algorithms to speed up the time of early diagnosis and ensure a better post-treatment outcome, in the case of diseases that are rare but have a prognostically inauspicious course if misrecognized until an advanced stage. It is hoped that the pandemic, which has now been efficiently controlled by public health services, and post-COVID-19 studies and research may help define a new route of approaching patients, especially oncology patients, for the purpose to improve the outcomes of some malignancies, such as the laryngeal adenoid cystic carcinoma.

**Table 2:** LACC diagnosis and treatment protocol.

		Findings	References
Diagnosis	Staging and histopathological assessment	It is necessary to perform an accurate histopathological analysis because histological grading seems to have a greater prognostic impact than TNM staging.	[11,12,13]
Treatment	Surgery	It is recommended to perform extensive surgical resection of the cancer. Total laryngectomy is the most used technique, partial laryngectomy can be used in selected cases.	[1,2,4,12,14,15,16,17,18,23]
	Adjuvant therapy	Adjuvant radiotherapy or chemoradiotherapy improves local control and survival. There is no direct comparison between protocols. Adjuvant therapy is particularly recommended in case of adverse features.	[1,13,16,24]
	Patients not eligible for surgery	Curative radiotherapy with radiotherapy with carbon ions (C12) has been used in few patients, with encouraging results. Further studies are needed.	[25,26]
Follow-up	Frequent follow-up	Frequent follow-up with whole-body evaluation is necessary, considering that cases of metastases have been reported in districts unusual for laryngeal carcinoma.	[8]

## Data Availability

The data presented in this study are available upon reasonable request from the corresponding author.
